# Activity of the cyclooxygenase 2-prostaglandin-E prostanoid receptor pathway in mice exposed to house dust mite aeroallergens, and impact of exogenous prostaglandin E_2_

**DOI:** 10.1186/1476-9255-6-30

**Published:** 2009-10-30

**Authors:** Aida Herrerias, Rosa Torres, Mariona Serra, Alberto Marco, Laura Pujols, César Picado, Fernando de Mora

**Affiliations:** 1 Department of Pharmacology, Universitat Autònoma de Barcelona, Barcelona, Spain; 2 Department of Pneumology and Respiratory Allergy, Hospital Clínic, IDIBAPS, Universitat de Barcelona, Spain; 3 CIBER [Centro de Investigación Biomédica en Red] de Enfermedades Respiratorias, Spain; 4 Department of Animal Pathology, Universitat Autònoma de Barcelona, Barcelona, Spain

## Abstract

**Background:**

Prostaglandin E_2 _(PGE_2_), experimentally administered to asthma patients or assayed in murine models, improves allergen-driven airway inflammation. The mechanisms are unknown, but fluctuations of the endogenous cyclooxygenase (COX)-2/prostaglandin/E prostanoid (EP) receptor pathway activity likely contribute to the clinical outcome. We analyzed the activity of the pathway in mice sensitized to aeroallergens, and then studied its modulation under exogenous PGE_2_.

**Methods:**

Mice were exposed to house dust mite (HDM) aeroallergens, a model that enable us to mimic the development of allergic asthma in humans, and were then treated with either subcutaneous PGE_2 _or the selective EP1/3 receptor agonist sulprostone. Simultaneously with airway responsiveness and inflammation, lung COX-2 and EP receptor mRNA expression were assessed. Levels of PGE_2_, PGI_2_, PGD_2 _were also determined in bronchoalveolar lavage fluid.

**Results:**

HDM-induced airway hyperreactivity and inflammation were accompanied by increased COX-2 mRNA production. In parallel, airway PGE_2 _and PGI_2_, but not PGD_2_, were upregulated, and the EP2 receptor showed overexpression. Subcutaneous PGE_2 _attenuated aeroallergen-driven airway eosinophilic inflammation and reduced endogenous PGE_2 _and PGI_2 _production. Sulprostone had neither an effect on airway responsiveness or inflammation nor diminished allergen-induced COX-2 and PGE_2 _overexpression. Finally, lung EP2 receptor levels remained high in mice treated with PGE_2_, but not in those treated with sulprostone.

**Conclusion:**

The lung COX-2/PGE_2_/EP2 receptor pathway is upregulated in HDM-exposed mice, possibly as an effort to attenuate allergen-induced airway inflammation. Exogenous PGE_2 _downregulates its endogenous counterpart but maintains EP2 overexpression, a phenomenon that might be required for administered PGE_2 _to exert its protective effect.

## Background

Allergic asthma is a common inflammatory disease of the airway, and long-term therapy is aimed at counteracting episodes of bronchospasm and reducing allergic inflammation. Although such strategies are successful, they neither cure nor prevent asthma, and, in some cases, have not prevented the disease from progressing [1]. Therefore, new therapeutic strategies must be identified [2]. Studies in mice models of asthma have shed light on the pathophysiology of the disease, and they have enabled us to hypothesize about novel targets for treatment [3,4]. The protective nature of endogenous molecules such as prostaglandin (PG) E_2 _provides us with an unusual opportunity to develop research projects aimed at uncovering novel targets. Interest in PGE_2 _as a clinically beneficial agent in asthma and asthma-like syndromes [5,6] has been rekindled in pre-clinical settings, and has encouraged investigators to further analyze the underlying mechanisms in vitro and in vivo [7-9]. The roles of endogenous PGE_2 _and of fluctuations in cyclooxygenase (COX)-2 activity in modulating airway reactivity and bronchial inflammation have been investigated in experimental rodent models of asthma by various groups [10,11], including ours [12,13]. In addition, we recently reported an improvement in airway inflammation after administration of subcutaneous PGE_2 _in the murine airway response to house dust mite (HDM) aeroallergens [14]. Our study reproduced observations in humans [5,6] and some of the very recently published data from ovalbumin (OVA)-sensitized mice [7]. Although little is known about the mechanisms involved, our work has also pointed to a PGE_2_-induced restraining effect on airway mast cell activity as a potentially relevant mediating phenomenon [13,14]. These in vivo data build on the results of in vitro experiments in which anti-inflammatory and immunosuppressive actions of PGE_2 _had been reported. PGE_2 _has been shown to exert an inhibitory effect on the activity of mast cells [8,15] and to suppress immunological mechanisms such as dendritic [16], and T [17] cell activation. The findings of our and other groups point to a protective effect of PGE_2 _involving several stages of asthma progression. A recurrent finding is the effect of PGE_2 _on cellular COX expression in vitro [18-20]. This is an area of interest, since the external provision of an endogenous molecule such as a PG might also affect in vivo the balance of the internal system of COX-2-PGE_2_-EP, and such an effect on the endogenous COX pathway possibly contributes to the clinical benefit resulting from administration of PGE_2_. Similarly, the PGE_2_-induced fluctuation of E prostanoid (EP) receptor (PGE_2 _receptor) expression shown in vitro [21,22], may have a profound impact on the ability of exogenous PGE_2 _to modulate the murine airway response to HDM. The EP3 receptor may be a main candidate for protection [23]. Despite its interest, the functional consequences on the COX-2-PGE_2_-EP receptor pathway of administering PGE_2 _in vivo remains largely unknown. This gap is probably partly attributable to the lack of accurate data on the fluctuating activity of the endogenous COX system in aeroallergen-induced asthma. Therefore, the direction, the relevance, and the implications of the fluctuations of different elements of the COX pathway need to be ascertained as a whole in an in vivo system.

We used HDM-sensitized mice, whose unique features enable us to mimic the development of allergic asthma in humans, to characterize in vivo the COX-2-PGE_2_-EP pathway. Under the hypothesis that PGE_2_-driven changes in airway inflammation are also attributable to fluctuations in the internal functioning of this axis, we proceeded to evaluate how expression of COX-2, PG, and EP were affected by the administration of PGE_2_.

## Methods

### HDM-sensitive mice and experimental groups

Samples from mice sensitized to HDM aeroallergens that had been shown to develop airway hyperreactivity and inflammation [14], were used in the present study. Briefly, eight-week-old female BALBc mice (Harlan, Spain) housed under a 12-hour light-dark cycle, had been exposed to a purified HDM extract (Alk-Abelló, Madrid, Spain) with a low lipopolysaccharide (LPS) content (<0.5 EU/dose, measured using the Charles River Endosafe Limulus Amebocyte Assay, Charles River Laboratories, Wilmington, Massachusetts, USA). The allergen was administered intranasally at a dose of 25 μg/mouse in a 10 μl volume for 10 consecutive days. Immediately before administration of HDM, light anesthesia was induced in a chamber filled with 4% halothane delivered over a period of 2 minutes in 100% oxygen and maintained for 2 additional minutes with 2.5% halothane. Non-sensitized (control) animals were handled identically, except that they received intranasal saline instead of HDM extract.

Six experimental groups were established. The first 3 groups contained non-sensitized (control) mice: group 1 contained untreated mice (n = 15) and groups 2 and 3 contained PGE_2_-treated (n = 15) and sulprostone-treated (n = 5) animals, respectively. The remaining 3 groups contained HDM-sensitized mice: group 4 included untreated animals (n = 21), and groups 5 and 6 contained PGE_2_-treated (n = 21) and sulprostone-treated (n = 11) animals, respectively. All animal procedures were approved by the Ethics Committee for Animal Research of the Universitat Autònoma de Barcelona.

### Administration of subcutaneous PGE_2 _and sulprostone

Both HDM-sensitized and non-sensitized mice had been treated with either PGE_2 _(0.5 mg/kg) or sulprostone (80 μg/kg), an EP3 agonist with a slight EP1 effect. Both EP receptor agonists were injected subcutaneously on the same day as the HDM extract, although their administration was continued up to day 11 (ie, 2 days after the allergen was withdrawn). The prostanoid treatment was provided 1 hour before exposure to HDM. PGE_2 _was purchased from Cayman (Tallinn, Estonia, ref. 14010) and the solution was prepared daily in phosphate-buffered saline (PBS) from a stock solution dissolved in dimethyl sulfoxide (DMSO) and stored at -20°C. The final concentration of DMSO injected was 0.1%. Sulprostone (Cayman, ref. 14765) was also prepared daily in PBS, and the solution administered contained 0.05% DMSO. The untreated mice underwent the same procedure, except that they received subcutaneous vehicle (PBS containing 0.1% DMSO) instead of the EP agonist.

### Assessment of COX-2 mRNA expression in the lungs

COX-2 mRNA expression in the lungs was assessed by real time polymerase chain reaction (PCR). After sacrifice, the intermediate lung lobe was kept at -80°C for RNA extraction. Total RNA was extracted using Trireagent (Molecular Research Center Inc, Cincinnati, Ohio, USA), and traces of contaminating genomic DNA were removed using DNAfree (Ambion Inc, Austin, Texas, USA). COX-2 cDNA was generated using MMLV reverse transcriptase (Epicentre, Madison, Wisconsin, USA). For real time-PCR, 2 μg of total RNA from each animal was reverse-transcribed and 2 μl of a 1/5 dilution of the resulting cDNA was placed into glass capillaries together with 18 μl of a master-mix. The COX-2 primers were designed with PrimerSelect software (DNASTAR Inc, Madison, Wisconsin, USA), and were as follows: forward primer 5'AGCCAGCAAAGCCTAGAGCAACAA3' and reverse primer 5'TGACCACGAGAAACGGAACTAAGAGG3'. PCR was performed in a LightCycler Instrument and the crossing point (CP, defined as the point at which fluorescence increases appreciably above background fluorescence) was determined by LightCycler software (both from Roche Diagnostics, Mannheim, Germany) using the second derivative maximum method.

### Assessment of PGE_2_, PGI_2 _and PGD_2 _levels in BAL fluid

After sacrifice, BAL fluid was centrifuged at 400 rcf for 5 minutes at 4°C and supernatants were collected and stored at -80°C for analysis. The endogenous production of PGE_2_, 6-keto PGF_1_α (a metabolite of PGI_2_), and PGD_2 _was determined in BAL fluid using a commercial competitive ELISA (Cayman, ref: 514010, 515211, 512021) following the manufacturer's instructions. Briefly, either the standards or the samples were incubated with the tracer antibody, the wells were then washed to remove all unbound reagents, and the signal was developed with Ellman's reagent.

### Assessment of mRNA expression EP1, 2, 3, and 4 receptor in the lungs

mRNA expression of EP receptors 1 to 4 in the lungs was assessed by real-time PCR using TaqMan^® ^Gene Expression Assays containing two unlabeled primers and one 6-FAM™ dye-labeled TaqMan^® ^MGB probe (Applied Biosystems, Foster City, California, USA, ref: Mm00443097_m1, Mm00436051_m1, Mm0.1316856_m1, Mm00436053_m1). Total RNA extraction and contaminating genomic DNA elimination were performed as for the assessment of COX-2 expression. EP1, 2, 3, and 4 cDNA was generated using MMLV reverse transcriptase (Epicentre, Madison, Wisconsin, USA). For real time-PCR, 2 μg of total RNA from each animal was reverse-transcribed and 4 μl of a 1/2 dilution of the resulting cDNA was placed into a 96-well reaction plate together with 16 μl of the master-mix. The real-time PCR reaction was run on a 7900 HT Real-Time PCR System (Applied Biosystems). The crossing point (CP, defined as the point at which fluorescence increases appreciably above background fluorescence) was determined by 7900 HT Real-Time PCR System software (Applied Biosystems) using the second derivative maximum method.

### Statistical analysis and calculations

As for the real time PCR results analysis, the Relative Expression Software Tool (REST^©^) was applied to calculate the relative expression ratio on the basis of group means for COX-2 or EP receptors (target genes) versus the reference gene GAPDH. The calculated group ratio was tested for significance using a statistical model known as the Pair Wise Fixed Reallocation Randomisation Test^© ^[24]. We took into account the PCR efficiency calculated for the target genes (COX-2 and EP1-4) and for GAPDH, which were very similar. As previously published [12], for purposes of graphic representation, the target genes (COX-2 and EP1-4) mRNA expression ratio of the untreated non-sensitized mice was established as 1.0, and the average ratios of the other experimental groups were re-calculated on that basis. ELISA PG levels were compared between groups using the *t *test.

## Results

### Fluctuation of COX-2 pathway activity in the lungs of HDM-sensitized mice

**Figure 1:**
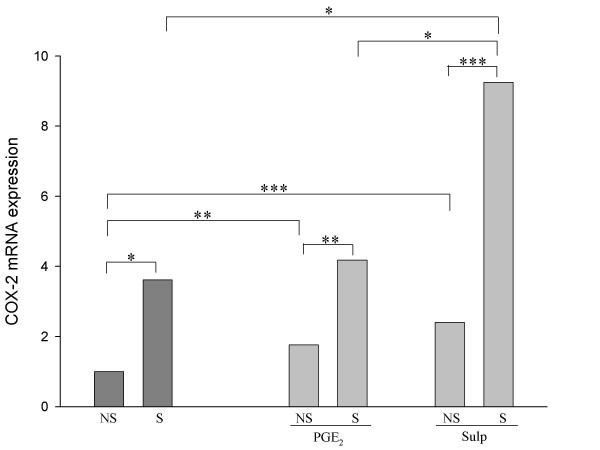
**Expression of COX-2 mRNA in the lung parenchyma as assayed by real-time PCR**. The relative mRNA expression ratio in the non-sensitized (and untreated) mice was established as 1.0. COX-2 mRNA expression in the lungs increased 3.6 fold in HDM-sensitized (n = 11) versus non-sensitized mice (n = 5). Baseline levels of COX-2 mRNA in the lungs were higher in non-sensitized mice under both PGE_2 _(n = 5) and sulprostone (n = 5) when compared to levels in non-sensitized and non-treated animals. COX-2 expression in PGE_2 _(n = 11) and sulprostone-treated mice (n = 11) increased by 2.5 and 3.8 fold, respectively, when the animals were exposed to HDM (*p < 0.05, **p < 0.01, ***p < 0.005).

**Figure 2:**
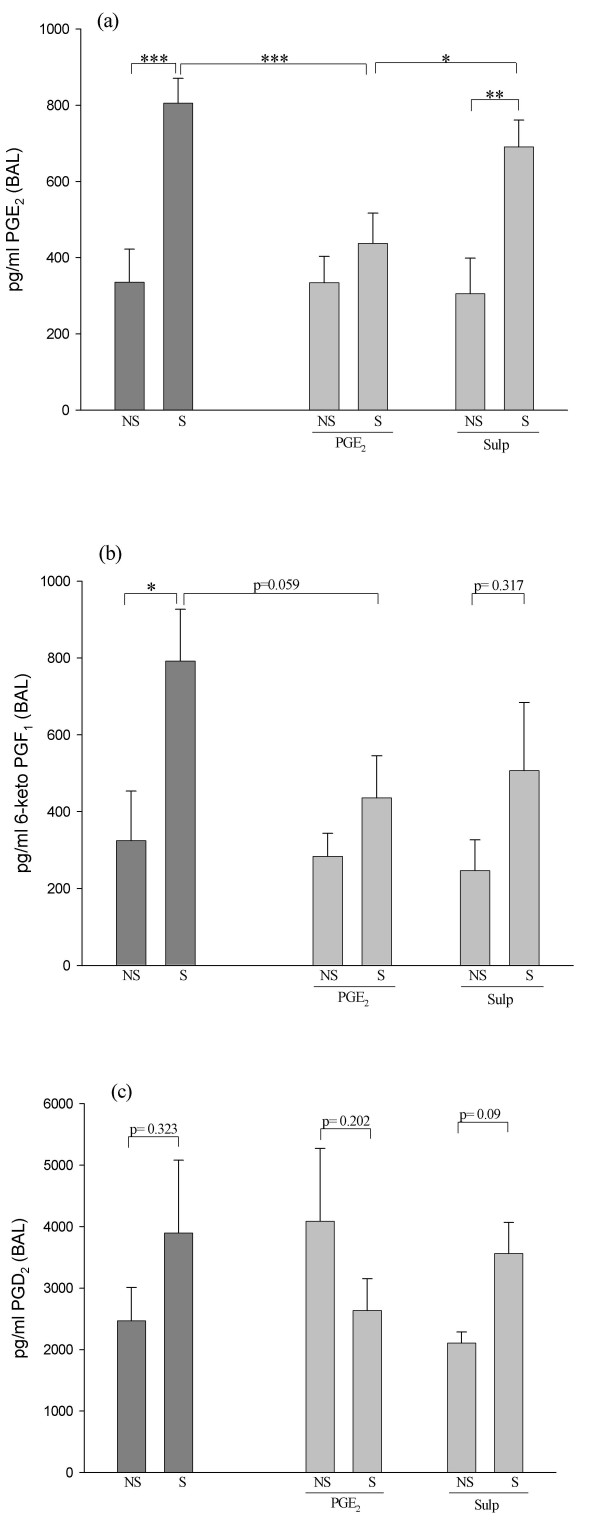
**Endogenous prostaglandin production in the airways as assayed by ELISA in BAL fluid**. Graph (a) shows endogenous PGE_2 _production. PGE_2 _increased 2.4 fold in allergen-sensitized (n = 11) versus non-sensitized mice (n = 5). Endogenous PGE_2 _production fell significantly to baseline levels in HDM-sensitized mice treated with PGE_2 _(n = 11), but remained unchanged when mice were treated with sulprostone (n = 11). Graph (b) depicts endogenous airway 6-keto PGF_1_α production (a metabolite of PGI_2_). In the same way as PGE_2_, 6-keto PGF_1_α increased 2.4 fold in allergen-sensitized (n = 11) versus non-sensitized mice (n = 5). 6-keto PGF_1_α production fell in HDM-sensitized mice treated with PGE_2 _(n = 11) and sulprostone had a similar inhibitory effect on BAL 6-keto PGF_1_α expression (n = 11). Graph (c) shows endogenous PGD_2 _production. No differences were found in BAL fluid levels of PGD_2 _in mice between any of the experimental groups (*p < 0.05, **p < 0.01, ***p < 0.005).

**Figure 3:**
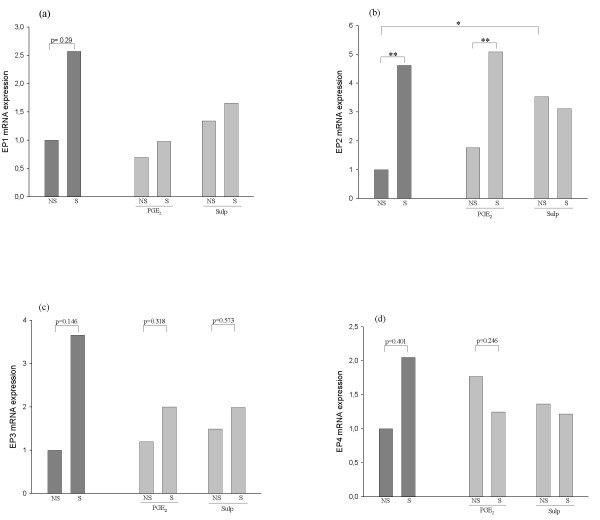
**Relative expression of EP 1, 2, 3 and 4 receptors mRNA in lung tissue assayed by real-time PCR**. Graphs a, b, c, and d show the mRNA expression of the EP1, EP2, EP3, and EP4 receptors, respectively, in the different treatment groups. EP receptor mRNA expression was higher for all four receptors in HDM-sensitized mice (n = 11) than in non-sensitized animals (n = 5), but only EP2 showed a significant allergen-induced upregulation (4.6 fold). Treatment with either agonist (PGE_2 _or sulprostone) did not significantly alter the level of expression of lung EP1, 3, and 4 in non-sensitized (n = 5) or HDM-sensitized mice (n = 11). However, sulprostone, but not PGE_2_, induced a 3.5-fold increased expression of EP2 baseline levels (non-sensitized mice), and it then prevented HDM from further enhancing these levels (*p < 0.05, **p < 0.01).

As shown previously, mice intranasally sensitized to HDM developed significant airway hyperreactivity and eosinophilic inflammation [14] when compared to non-sensitized animals. This reaction was accompanied by changes in the local expression of COX-2, PG, and EP receptors, as depicted by the dark grey bars in Figures 1 through 3. All these COX-2 pathway molecules were determined simultaneously in every single animal, and measurements were performed 48 hours after the last challenge with HDM. COX-2 mRNA expression in the lungs increased 3.6 fold in mice sensitized to HDM aeroallergens compared with non-sensitized mice (Figure 1). COX-2 overexpression in the airways of HDM-sensitized animals was accompanied by a 2.4-fold increase in the production of both PGE_2 _(Figure 2a) and PGI_2 _(Figure 2b), but not PGD_2 _(Figure 2c) in BAL fluid. The mRNA expression of PGE_2 _receptors EP 1 to 4 was also determined in lung extracts (Figure 3). Despite higher levels of mRNA in all four receptors in HDM-sensitized mice than in non-sensitized mice, only EP2 showed a statistically significant allergen-induced upregulation in HDM-sensitized mice - 4.6-fold (Figure 3b).

### Effect of exogenous PGE_2 _on airway COX-2 and PG expression in HDM-sensitized and non-sensitized mice

As previously reported [14], subcutaneous PGE_2_, but not sulprostone, significantly reduced HDM-induced eosinophil recruitment into the airways (by approximately 40%), but had no effect on methacholine-induced airway hyperreactivity. In these animals, the effect of exogenous PGE_2 _and sulprostone on airway COX-2 activity was measured by evaluating COX-2 mRNA expression in parallel to the BAL COX-2 products PGE_2_, PGI_2_, and PGD_2_. Baseline levels of lung COX-2 mRNA in non-sensitized mice were significantly increased under both the PGE_2 _(1.8-fold increase) and the sulprostone (2.4-fold increase) treatment when compared with levels in non-sensitized or non-treated animals (Figure 1). As observed in non-treated sensitized animals, COX-2 expression in PGE_2_- and sulprostone-treated mice increased when these mice were sensitized to HDM allergens. The magnitude of this increase was 2.5 fold and 3.8 fold for mice under PGE_2 _and sulprostone, respectively.

As for airway COX-2 product synthesis, HDM-induced enhanced endogenous PGE_2 _production returned to baseline values in sensitized animals treated with exogenous PGE_2_. This effect was uncovered by the significant difference in PGE_2 _levels in BAL between sensitized non-treated and PGE_2_-treated mice (Figure 2a). However, sulprostone did not reduce endogenous PGE_2 _production in HDM-sensitized mice. Similarly, the increase in PGI_2 _in BAL in HDM-sensitized mice was lower after administration of external PGE_2 _(Figure 2b). Sulprostone had a similar inhibitory effect on PGI_2 _production. Finally, PGD_2 _was not significantly affected by treatment with either agonist (Figure 2c).

### Effect of exogenous PGE_2 _on EP receptor expression in the lungs of HDM-sensitized mice

Figure 3a, b, c, and 3d depict the effect of PGE_2 _and sulprostone on lung EP receptors 1, 2, 3, and 4 mRNA expression, respectively. Treatment with either agonist did not significantly alter the level of expression of lung EP1, 3, or 4 in either baseline (non-sensitized) or HDM-sensitized mice from a statistical perspective. However, as for HDM-induced EP2 overexpression in sensitized mice, sulprostone, but not PGE_2_, prevented upregulation at 2 different levels: it induced a 3.5-fold increase in the baseline expression of EP2, and it prevented HDM from further enhancing this baseline level.

## Discussion

We have shown that, in addition to inducing airway hyperreactivity and eosinophil recruitment, intranasal exposure to HDM alters the endogenous COX-2 pathway at various levels: it upregulates COX-2, PGE_2_, and PGI_2 _in the lungs, and it enhances local EP_2 _receptor expression. Exogenous PGE_2 _modulates these HDM-induced changes in the COX2/PG/EP receptor pathway. Notably, in addition to reducing airway eosinophilia, it prevents HDM-induced lung PGE_2 _and I_2 _overexpression, but does not counteract HDM aeroallergens-induced EP_2 _upregulation.

Lung COX-2 mRNA expression and generation of its product PGE_2_, are increased in HDM-sensitized mice. Interestingly, a similar pattern is observed with PGI_2_, another anti-inflammatory PG [25], but not with PGD_2_. We know that COX-2 upregulation is tightly linked to PGE synthase (PGES-1) activity [26]. In turn, although PGI_2 _was traditionally considered to derive mainly from COX-1, in the last few years this paradigm has proven to be incorrect, since studies in mice and humans have shown that COX-2 is the dominant source of PGI_2 _[26]. The fluctuations of PGE_2 _and PGI_2 _are therefore possibly the result of enhanced expression of COX-2. As far as we know, ours is the first report to demonstrate aeroallergen-induced modulation of the complete COX-2 pathway. This builds on our previous results [12], in which we detected a trend towards increased COX-2 and PGE_2 _activity in the lungs of HDM-sensitized mice, and on an earlier report in which OVA-sensitized guinea pigs were used [27]. It is difficult to uncover the significance of aeroallergen-induced increased lung COX-2 production. According to our and others hypothesis, asthma develops partly as a result of improper regulation of COX-2 activity [28,29], and, given that PGE_2 _and PGI_2 _are considered anti-inflammatory protective prostanoids within the lungs [25,30], we speculate that PGE_2 _and PGI_2_, but not PGD_2_, attempt to trigger a beneficial compensatory phenomenon in mice exposed to HDM. Although this concept has yet to be proven, our in vivo data confirm in vitro findings where the overactivity of the COX-2/PGE_2_/EP_2 _pathway was viewed as an effort to minimize allergen-induced damage [18,31]. This idea is reinforced by the fact that airway pathology worsens when endogenous PGE_2 _is presumably inhibited either genetically or pharmacologically in antigen-sensitized mice, as shown by ours [13] and other groups [10,11].

PGD_2_, in turn, has traditionally been described as a PG inducing functional exacerbation of the airways, despite the fact that recent articles report a potentially beneficial effect [32,33]. In our view, the unchanged levels of PGD_2 _in our setting two days after the last allergen challenge could be attributable to timing issues, i.e. PGD_2 _probably peaks immediately after challenge. If so, a different experimental time-course approach would be required to reveal such PGD_2 _fluctuations in the HDM mouse model.

Data on whether COX-2, PGE_2 _and/or PGI_2 _are overproduced or not in the lungs of asthmatics are contradictory. Several authors have described either upregulation, or downregulation and even unchanged levels [28,29,34-37]. These contradictions could be timing-related [38] or be genetically determined; in any case, they reflect the complexity of understanding COX-2/PG dynamics in the lungs of asthmatics and confirm the need for an experimental in vivo approach to identify the actual changes and their clinical impact. The genetic basis is a fundamental issue, since it has been hypothesized that the COX-2 gene might be altered in asthmatics [28,29,38]. This potential human genetic defect does not affect mice and this probably explains why in our study the animals remain fully able to respond with consistent COX-2 activity increases when exposed to aeroallergens.

In addition to COX-2 and PGE_2_, intranasal HDM selectively increases EP2 receptor expression in the lungs of mice. It is worth noting that increases in mRNA levels were detected in all four receptors, but that statistical significance was only reached with EP2. The lack of statistical significance in EP1, 3 and 4 is probably attributable to interindividual variability of endogenous molecules expression and to the nature of the mRNA detection system. Despite such technical limitations, EP2 overexpression was shown to be consistent and statistically significant. This would suggest that EP2 uregulation is a relevant trait of an internal defensive strategy of the COX-2/PGE_2_/EP pathway against HDM aeroallergens aggression, but such statement requires further experimental evidence. Our hypothesis on a leading anti-inflammatory role of EP2 in HDM-sensitive mice would agree with findings from in vitro experimental approaches where EP2 was proposed a candidate receptor to mediate the beneficial effects of PGE_2 _in humans [7,8,15]. Although not reported from in vivo experiments in mice models, a selective upregulation of EP2 has been described by Burgess JK et al. [31] in airway smooth muscle cells from asthmatics. All in all, an internal EP2-mediated compensatory mechanism aimed at reducing the damage induced by HDM in animals whose COX-2/PG armamentarium is genetically intact seems to be a reasonable explanation. In order to ascertain the relevance of a selective EP2 increase in attenuating airway pathology, EP2 receptor genetic manipulation (e.g. antisense oligonucleotide or iRNA) would be required.

A recent report by our group [14] showed that PGE_2 _significantly reduced to almost a half HDM-induced airway eosinophilia, but had no effect on AHR. An intensive single-dose treatment protocol with the agonists starting a day before the actual exposure to HDM was used with the purpose of ensuring that effective prostanoid levels would be present during the relevant phases of the process, regardless of the clinical relevance of such concentrations. The early treatment with the EP agonists was also partly based on the reported immuosuppressive effects of PGE2 in vitro [16,17]. We have now observed that, under this protocol, in parallel to preventing eosinophil recruitment, exogenous PGE_2 _clearly attenuates endogenous production of PGE_2 _and PGI_2_. Lung COX-2 expression, if at all, is only very slightly affected and certainly not to the extent of the change in PG production. A straightforward explanation would be that exogenous PGE_2 _overtakes the role exerted by the endogenous PG, with no need to maintain a similar endogenous production of PGE_2 _and PGI_2_, since an external source of PGE_2 _is already provided. This would therefore be viewed as a classical negative feedback mechanism, possibly on the PG synthases rather than COX-2. Alternatively, the reduced PG expression in the presence of exogenous PGE2 might be the result of less infiltrated inflammatory cells producing such PG within the airways.

Sulprostone neither reduces inflammation nor alters the HDM-induced increase in COX-2 or PGE_2 _levels. It does exert some effect on the level of COX-2 mRNA expression, although such an effect is similar in HDM-sensitized and non-sensitized animals. Therefore, this phenomenon does not selectively occur in allergen-sensitized mice. This somehow shows that EP1/EP3 and EP2 (and possibly EP4) are independent systems, and confirms that PGE_2 _anti-inflammatory activity in HDM aeroallergens-induced airway pathology in mice is more likely the result of an EP2-mediated effect as discussed earlier. To confirm this hypothesis further experiments with an EP2 selective agonist are required. Interestingly, the analysis of airway EP receptor expression in the presence of EP receptor agonists brings us to a similar conclusion. Exogenous PGE_2 _does not prevent the HDM-induced increase in EP2, but sulprostone does. Given the observed anti-inflammatory nature of PGE_2 _(but not sulprostone) [14], this supports the assertion that the increase in EP2 is necessary in mediating the anti-inflammatory effect of PGE_2_. Furthermore, our data suggest that an EP2 agonist, whether exogenous or endogenous, is needed to keep EP2 levels raised. Finally, it is noteworthy that lung levels of EP2 are similar in HDM-sensitized mice regardless whether they are treated or not with PGE_2_, and yet PGE_2_-treated mice do have lower numbers of eosinophils in the airways [14]. This suggests that exogenously delivered PGE_2 _peaks (undetected by ELISA) are necessary for protection simultaneously to the overexpression of EP2.

## Conclusion

In summary, we can infer that exposure to HDM aeroallergens in mice boosts the COX-2-PGE_2_-EP2 pathway, possibly to alleviate progression of asthma. This effect counterbalances HDM-induced damage by selectively incrementing the interaction of PGE_2 _with its EP2 receptor. The exogenous provision of PGE_2 _precludes endogenous counterparts from augmenting but helps sustain high levels of EP2. This is the first report to characterize the complete lung COX-2 pathway in vivo in a mouse model of asthma including enzyme expression, PG production, and PGE_2 _receptor expression. Given the complexity of the multiple effects of PG, a time-course variable needs to be incorporated into such studies to assess the fluctuating activity of the endogenous COX-2 pathway in HDM-sensitized mice, whether treated with PGE_2 _or not, with the final aim of proposing potential targets for pharmacological development.

## Competing interests

The authors declare that they have no competing interests.

## Authors' contributions

FDM obtained funding for the project, provided overall guidance for the study, assisted in the analysis and interpretation of the data, and prepared the manuscript. AH participated in the experimental design, planned and performed all of the experiments, and helped in the writing of the manuscript. RT, MS, and LP participated in sample and data collection, and helped in the revision of the manuscript. CP participated in the acquisition of funding, designing the experiments, and revising the manuscript. AM contributed to the design of the experimental approach and revised the manuscript. All the authors have read and approved the final manuscript.
